# Identifying Antibiotic Prescribing Patterns Through Multi-Level Latent Profile Analyses: A Cross-Sectional Survey of Primary Care Physicians

**DOI:** 10.3389/fphar.2020.591709

**Published:** 2020-11-11

**Authors:** Dan Wang, Chaojie Liu, Xinping Zhang, Chenxi Liu

**Affiliations:** ^1^ School of Medicine and Health Management, Tongji Medical School, Huazhong University of Science and Technology, Wuhan, China; ^2^ School of Psychology and Public Health, La Trobe University, Melbourne, VIC, Australia

**Keywords:** primacy care, antibiotic prescription, latent profile analysis, knowledge-attitudes-practices, China

## Abstract

**Background**: Overuse of antibiotics significantly fuels the development of Antimicrobial resistance, which threating the global population health. Great variations existed in antibiotic prescribing practices among physicians, indicating improvement potential for rational use of antibiotics. This study aims to identify antibiotic prescribing patterns of primary care physicians and potential determinants.

**Methods**: A cross-sectional survey was conducted on 551 physicians from 67 primary care facilities in Hubei selected through random cluster sampling, tapping into their knowledge, attitudes and prescribing practices toward antibiotics. Prescriptions (*n* = 501,072) made by the participants from 1 January to March 31, 2018 were extracted from the medical records system. Seven indicators were calculated for each prescriber: average number of medicines per prescription, average number of antibiotics per prescription, percentage of prescriptions containing antibiotics, percentage of antibiotic prescriptions containing broad-spectrum antibiotics, percentage of antibiotic prescriptions containing parenteral administered antibiotics, percentage of antibiotic prescriptions containing restricted antibiotics, and percentage of antibiotic prescriptions containing antibiotics included in the WHO “Watch and Reserve” list. Two-level latent profile analyses were performed to identify the antibiotic prescribing patterns of physicians based on those indicators. Multi-nominal logistic regression models were established to identify determinants with the antibiotic prescribing patterns.

**Results**: On average, each primary care physician issued 909 (ranging from 100 to 11,941 with a median of 474) prescriptions over the study period. The mean percentage of prescriptions containing antibiotics issued by the physicians reached 52.19% (SD = 17.20%). Of those antibiotic prescriptions, an average of 82.29% (SD = 15.83%) contained broad-spectrum antibiotics; 71.92% (SD = 21.42%) contained parenteral administered antibiotics; 23.52% (SD = 19.12%) contained antibiotics restricted by the regional government; and 67.74% (SD = 20.98%) contained antibiotics listed in the WHO “Watch and Reserve” list. About 28.49% of the prescribers were identified as low antibiotic users, compared with 51.18% medium users and 20.33% high users. Higher use of antibiotics was associated with insufficient knowledge, indifference to changes, complacency with satisfied patients, low household income and rural location of the prescribers.

**Conclusion**: Great variation in antibiotic prescribing patterns exists among primary care physicians in Hubei of China. High use of antibiotics is not only associated with knowledge shortfalls but also low socioeconomic status of prescribers.

## Introduction

Antimicrobial resistance (AMR), one of the most alarming threats to global health, has resulted in significant human and economic loss worldwide. It was estimated that AMR led to 700,000 deaths in 2014 (O’Neill, 2014). Without effective interventions, this figure would balloon to 10 million per year by 2050 and become the main cause of death globally (O’Neill, 2014).

Overuse of antibiotics is widely believed to be associated with the development of AMR (Bronzwaer et al., 2002; Goossens et al., 2005; Hay et al., 2005; van de Sande-Bruinsma et al., 2008). Given that the discovery of new antibiotics has been dramatically slow over the past few decades (Llor and Bjerrum, 2014), reducing irrational prescriptions of antibiotics became an urgent public health agenda. Unfortunately, irrational antibiotic prescribing has been prevalent worldwide. In the US, for example, over 50% antibiotic prescriptions are deemed inappropriate and 30% unnecessary (Shapiro et al., 2014).

Past studies revealed that great variations existed in antibiotic prescribing practices among physicians (Jones et al., 2015; Aabenhus et al., 2017; Pouwels et al., 2018; Schmidt et al., 2018; Jung et al., 2019; Schwartz et al., 2019), which could not be fully explained by the variation in patient needs (Pouwels et al., 2018; Schwartz et al., 2019). The disparity of antibiotic prescription rates among prescribers could be as high as ten times after casemix adjustments for patients (Jung et al., 2019). A recent study in Canada demonstrated that the same patient would have 1.7 times more or less chance to receive antibiotics simply by swapping to a different physician (Schwartz et al., 2019).

Internationally, both restrictive and persuasive measures have been attempted to curtail irrational antibiotic prescribing behaviors (van der Velden et al., 2012; Drekonja et al., 2015; Pinder et al., 2015; Köchling et al., 2018). But very few interventions, if any, have tailored to the individual differences across prescribers. This could seriously jeopardize the efficiency and effectiveness of the interventions (van der Velden et al., 2012; Drekonja et al., 2015; Köchling et al., 2018).

This study aimed to identify individual antibiotic prescribing patterns in primary care physicians through latent profile analyses, a method that categorizes prescribing behaviors using multiple indicators. Our current understanding about antibiotic prescribing patterns is quite limited (Lopez-Vazquez et al., 2012; Rodrigues et al., 2013). Previous studies often adopted an over-simplified approach by examining the frequency and volume of antibiotics prescribed (Jones et al., 2015; Aabenhus et al., 2017; Pouwels et al., 2018; Schmidt et al., 2018; Jung et al., 2019; Schwartz et al., 2019). Such kind of study, although important, has failed to reveal the complex nature of antibiotic prescribing behaviors. Theoretically, irrational prescribing of antibiotics can also be reflected through the type of antibiotics (e.g., narrow-vs broad-spectrum) and the way they are administered (e.g., oral vs. parenteral) (World Health Organization, 2019). This study fills the gap in the literature by employing latent profile analyses, which can help identify irrational antibiotic prescribers who would otherwise be missed in single indicators.

## Participants and Methods

### Setting

The study was conducted in Hubei province of central China. Hubei has a land size of 185,900 km^2^ and more than 59 million populations. With a gross domestic product at $8,915 per capita in 2017, its economic status is ranked in the middle range of all provinces in China. According to the World Bank (National Bureau of Statistics of China, 2018), Hubei is considered as a middle-high income region.

We chose primary care facilities in Hubei as the study setting. In 2017, primary care facilities in Hubei served 205.08 million patient visits, accounting for 60.24% of total outpatient visits in the province (Hubei Government, 2017). About 44.28% of the patient visits to primary care were given an antibiotic prescription (Liu et al., 2019a). 60% primary care antibiotic prescriptions was estimated as inappropriate in China (Wang et al., 2014).

Since there is no well-established real-time dynamic surveillance system of antibiotic use in primary cares in Hubei, antibiotic use in these facilities was monitored based on annual or research report, in which the amount of antibiotic use was revealed (Hubei Government, 2017). Governments also help regulate antibiotic use by publishing policies, including recommending percentage of antibiotic use in primary cares (20%), issuing a list of restricted antibiotics for primary cares etc. (Hubei Provincial Health Committee, 2018; Liu et al., 2019a). However, whether and to what extent these policies were implemented into practices among primary cares is still unclear.

### Sampling and Participants

A stratified cluster random sampling strategy was applied to generate study participants. Hubei has 347 urban community health centers and 1,137 rural township health centers. In proportion to the urban and rural numbers, 19 community healthcare centers from three urban districts and 48 township health centers from six rural districts were randomly selected, respectively. Details of the sampling methods have been published elsewhere (Liu et al., 2019a).

Primary care physicians from the selected health centers who met the following criteria were invited to participate in the study: 1) having the authority to independently prescribe antibiotics; 2) having issued at least 100 prescriptions during the three-month study period, which contained at least one antibiotic prescription.

In total, 645 physicians met the inclusion criteria and 551 (85.58%) agreed to participate in the study. Of those who agreed, 458 (71.01%) returned a valid questionnaire.

### Data Collection

Eight field investigators were recruited and trained to conduct data collection. A pair of the trained investigators visited each participating facility. The investigators had no servicing relationships with the facilities or their employees at the time. All eligible primary care physicians were approached and invited to participate in the study. Informed written consents were obtained prior to data collection.

Prescribing records issued by the 551 study participants over a three-month period (1 January–31 March 2018) were extracted from the medical records system of the participating facilities, including the name, formulation, dosage, administration route, and price of the prescribed medicines, and information about the prescribers and facilities. This was followed by a questionnaire survey of prescribers (*n* = 458) during 23 April to June 6, 2018, tapping into their socioeconomic status and professional characteristics, and their knowledge and attitudes toward antibiotic prescribing. The respondents were asked to complete the questionnaire independently, which took roughly 15 min. A token gift ($1.65) was given to those who returned the questionnaire to the investigators. Missing items, if any, were re-filled by the investigators through an additional interview.

### Measurements

#### Antibiotic Prescribing Patterns

Seven indicators were identified for measuring antibiotic prescribing patterns through a comprehensive literature review and expert consultations:

- average number of medicines issued per prescription;
- average number of antibiotics issued per prescription;
- percentage of prescriptions involvingantibiotics;
- percentage of antibiotic prescriptions involving broad-spectrum antibiotics;
- percentage of antibiotic prescriptions involving parenteral administered antibiotics;
- percentage of antibiotic prescriptions involving restricted antibiotics imposed by the provincial government; and
- percentage of antibiotic prescriptions involving antibiotics included in the World Health Organization (WHO) “Watch and Reserve List.”

The first three indicators were adapted from the prescribing indicators recommended by the WHO (Desalegn, 2013). They measured the frequency and volume of antibiotics prescribed. Although we did not measure combined use of antibiotics directly because it was rare in primary care, the tendency of combined use of antibiotics was likely to be captured through the connection between the volume (indicator 2) and frequency (indicator 3) indicators (Chem et al., 2018). Previous studies showed that higher number of medicines prescribed in general is also a significant predictor of higher antibiotic prescriptions (Lukali and Michelo 2015; Amaha et al., 2019). Due to insufficient quality of information system in primary cares and limited usefulness of current antibiotic use guideline, the quality of antibiotic use, for example, rational use of antibiotics adherence to guidelines, is not used (Niaz et al., 2019). Though there is a clinical guideline for antibiotic use in China (2015 version) (Working Group of Revision of Clinical Guidelines for Application of Antibacterial Agents, 2015), this guideline illustrates recommended treatment for clear diagnosis of bacterial infection, for example, bacterial pneumonia, bacterial meningitis etc. It is not the situation that physicians in primary cares facing in routine practices, in which limited diagnostic technique available, diagnostic uncertainty common and decision could not be made due to infections unable to be distinguished by viral or bacterial (Lum et al., 2018).

We added two additional indicators (indicator 4 and 5) in order to better assess irrational prescribing of antibiotics. Empirical evidence shows that broad-spectrum antibiotics is frequently used and is perhaps the most common form of antibiotic abuse in primary care (Wei et al., 2017; Zhang et al., 2017). In addition, the high prevalence of parenteral administration of antibiotics has attracted increasing safety concerns in China. Studies showed that 36%–60% of antibiotics were administered through parenteral injections in primary care settings in China (Wei et al., 2017; Zhang et al., 2017; Zhang et al., 2019), which has also been showed in the use of injectable proton pump inhibitors induced by financial incentives (Zeng et al., 2015).

Over the past two decades, China introduced some restrictive measures to reduce irrational antibiotic prescribing. These included a list of restricted antibiotics for primary care imposed by the regional governments (Hubei Provincial Health Committee, 2018). Restricted access to certain antibiotics addresses the concerns of AMR (Coenen et al., 2009; Sarpong and Miller, 2015; Hsia et al., 2019). The WHO also published an “Access, Watch and Reserve” (AWaRe) classification system (World Health Organization, 2015). All antibiotics were exclusively classified into three categories. The “Watch” list includes antibiotics that have higher resistance potential, while the “Reserve” list includes antibiotics that should be reserved for treatment of infections due to multi-drug-resistant organisms. We examined prescriptions of restricted antibiotics against the above two classification systems. Although the two share similar principles, they are not always consistent. In Hubei, antibiotics were classified into non-restricted, restricted, and special-restricted.

#### Factors Associated With Antibiotic Prescribing Patterns

Antibiotic prescribing behaviors can be influenced by the knowledge and attitudes of prescribers, their personal circumstances, availability of guidelines and influence of pharmaceutical companies, especially in LMICs. (Lopez-Vazquez et al., 2012; Rodrigues et al., 2013; Md Rezal et al., 2015; Riaz et al., 2015; Ogunleye et al., 2019). Prescribers with higher qualifications and better knowledge of antibiotics are less likely to prescribe antibiotics. However, their attitudes toward antibiotic prescribing are also influenced by patient expectations and collegial pressures.

This study used a 37-item questionnaire to measure the knowledge, attitudes and personal circumstances of prescribers. The questionnaire was developed based on some existing instruments (Liu et al., 2019b; Teixeira Rodrigues et al., 2016) with further consideration of the findings of the two systematic reviews (Lopez-Vazquez et al., 2012; Rodrigues et al., 2013). The questionnaire reliability and validity has been tested and confirmed in previous studies (Liu et al., 2019b; Teixeira Rodrigues et al., 2016).

The questionnaire respondents were asked to indicate whether they agreed to prescribe antibiotics for 11 common conditions such as upper respiratory tract infections and diarrhea (Liu et al., 2019b). A correct decision in line with the current clinical guidelines was given a score of 1, otherwise 0. The scores were summed up for each respondent.

The attitudes of the questionnaire respondents toward antibiotic prescribing were assessed by 17 items, coded as a 5-point Likert scale (0 = strongly agree, 1 = agree, 2 = neutral, 3 = disagree, 4 = strongly disagree). The scores were summed up to measure the tendency of complacency to satisfied patients (0–8 measured by four items), fearful of adverse events (0–12 measured by six items), ignorance of AMR (0–16 measured by eight items), indifference to changes (0–4 measured by two items), and responsibility avoidance by blaming others (0–28 measured by seven items), respectively (Rodrigues et al., 2016). All item coding and summed scores were aligned into a unified direction, with a higher score indicating more positive attitudes toward reduction of irrational antibiotic prescribing.

The personal circumstances measured in this study included the demographic characteristics (age and gender) of the respondents, and their socioeconomic status (educational qualifications, and household income) and professional experiences (workplace, years of practice, sub-specialty, professional title, and continuing education on antibiotic prescribing). These factors have been proved to be significant determinants of antibiotic prescribing behaviors (Lopez-Vazquez et al., 2012; Rodrigues et al., 2013).

### Data Analysis

Two datasets were prepared for data analyses. The first dataset contained 501,072 prescriptions made by 551 primary care physicians. For each physician, the seven prescription indicators were calculated. Antibiotics were defined according to the Anatomical Therapeutic Chemical (ATC) classification system and included only systemic use of antibiotics (ATC code J01) (World Health Organization, 2019). They were further divided into broad- and narrow-spectrum in line with the classification criteria used in the US national survey on antibiotic use (Sarpong and Miller, 2015). Restricted antibiotics were defined based on the Hubei government’s antibiotic regulation policy and the WHO AWaRe list.

To determine the antibiotic prescribing patterns, latent profile analyses (LPA) were performed using the seven prescribing indicators at the physician level. LPA belong to finite mixture modeling which can identify and describe “hidden groups” within a population. Because the 551 physicians were clustered in 67 primary care facilities, a two-level LPA model was established. Differences at the facility level were treated as random effect. Maximum likelihood parameter estimates with standard errors (MLR) were applied. The model identification was checked using 1,000 initial stage starts and 1,000 final stage starts (Nylund-Gibson and Choi, 2018).

We tested different models that categorized antibiotic prescribing behaviors into one, two, three, four, or five groups. The best fit model was identified using the following model index: Bayesian Information Criterion (BIC), Sample-size Adjusted BIC (SABIC), Vuong-Lo-Mendell-Rubin Adjusted Likelihood Ratio Test (VLMR-LRT), Correct Model Probability (cmP) and Entropy. A lower value of BIC and SABIC indicates better fitness of data into the estimated model. VLMR-LRT compares the model fit between two neighboring models (for example, two groups vs three groups). A non-significant *p* value (>0.05) represents a lack of statistical significance between the two compared models. cmP provides an overall assessment of all estimated models and a larger cmP value indicates a better model fit. Entropy assesses the accuracy of classification, with a higher value indicating better classification (Nylund-Gibson and Choi, 2018). To avoid over-stratification, the smallest group should have a minimum of 5% of participants.

The second dataset contained the 458 returned questionnaires, as well as the classification of the antibiotic prescribing patterns of the 458 respondents. A three-group model was identified in the LPA. Each questionnaire respondent was assigned into one of the antibiotic prescribing pattern groups with the highest probability.

Differences in knowledge and attitudes scores and personal circumstances among the respondents in different antibiotic prescribing pattern groups were examined using Kruskal-Wallis rank tests, one-way analysis of variance (ANOVA), or chi-square tests. Post-hoc pairwise comparisons were performed using Dunn and Bonferroni tests. Multi-nominal logistic regression models were applied to determine significant factors predicting the three groups of antibiotic prescribing patterns after adjustments for variations in other factors. In the regression analyses, knowledge and attitudes scores were transformed into dichotomous variables with mean scores serving as a cut-off point. An enter approach was adopted in the modeling.

The statistical analyses were conducted using STATA (version 12.0) as well as Mplus (version 6.0). A *p* value < 0.05 was treated statistically significant.

## Results

### Antibiotic Prescribing in Primary Care

On average, 909 (ranges: 100–11,941, median: 474) prescriptions were issued by the 551 participating physicians over the three-month study period. Each physician prescribed an average of 2.87 (SD = 0.78) medicines and 0.65 (SD = 0.26) antibiotics per prescription, respectively. Of the prescribed antibiotics, cephalosporins (J01D) was the most commonly used (38.50%), followed by macrolides (J01F, 24.03%).

The mean percentage of prescriptions involving antibiotics prescribed by the physicians was 52.19% (SD = 17.20%). Of those prescriptions containing antibiotics, an average of 82.29% (SD = 15.83%) involved broad-spectrum antibiotics; 71.92% (SD = 21.42%) involved parenteral administered antibiotics; 23.52% (SD = 19.12%) involved restricted antibiotics imposed by the provincial government; and 67.74% (SD = 20.98%) involved antibiotics listed in the WHO “Watch and Reserve” list (Table 1).

**TABLE 1:** Prescribing patterns of primary care physicians.

Prescribing indicators	Mean ± standard deviation	Low antibiotic user (*n* = 157)	Medium antibiotic user (*n* = 282)	High antibiotic user (*n* = 112)	*p* value^a^			
					Low vs. medium	Low vs. high	Medium vs. high	Overall
Q1: Average number of medicines issued per prescription (*N*)	2.870 ± 0.775	2.334 ± 0.596	2.861 ± 0.655	3.645 ± 0.612	<0.001	<0.001	<0.001	<0.001
Q2: Average number of antibiotics issued per prescription (*N*)	0.654 ± 0.256	0.433 ± 0.171	0.636 ± 0.150	1.011 ± 0.170	<0.001	<0.001	<0.001	<0.001
Q3: Percentage of prescriptions involving antibiotics (%)	52.19 ± 17.20	36.76 ± 12.73	51.94 ± 11.29	74.43 ± 8.96	<0.001	<0.001	<0.001	<0.001
Q4: Percentage of antibiotic prescriptions involving broad-spectrum antibiotics (%)	82.29 ± 15.83	69.14 ± 17.77	87.09 ± 10.41	88.66 ± 13.41	<0.001	<0.001	0.031	<0.001
Q5: Percentage of antibiotic prescriptions involving parenteral administrated antibiotics (%)	71.92 ± 21.42	45.83 ± 17.35	79.7 ± 11.50	88.89 ± 10.48	<0.001	<0.001	<0.001	<0.001
Q6: Percentage of antibiotic prescriptions involving antibiotics in the WHO “watch and reserve” list (%)	67.74 ± 20.98	63.87 ± 20.56	66.85 ± 19.82	75.42 ± 22.60	0.232	<0.001	<0.001	<0.001
Q7: Percentage of antibiotic prescriptions involving restricted antibiotics (%)	23.52 ± 19.12	18.99 ± 16.94	25.19 ± 18.50	25.66 ± 22.37	0.001	0.042	0.718	0.002

a ANOVA and post-hoc pairwise Bonferroni tests for the indicators with a normal distribution; Kruskal-Wallis equality-of-populations rank tests and post-hoc pairwise Dunn’s tests for the indicators without a normal distribution.

### Primary Care Physicians’ Antibiotic Prescribing Patterns

**FIGURE 1:**
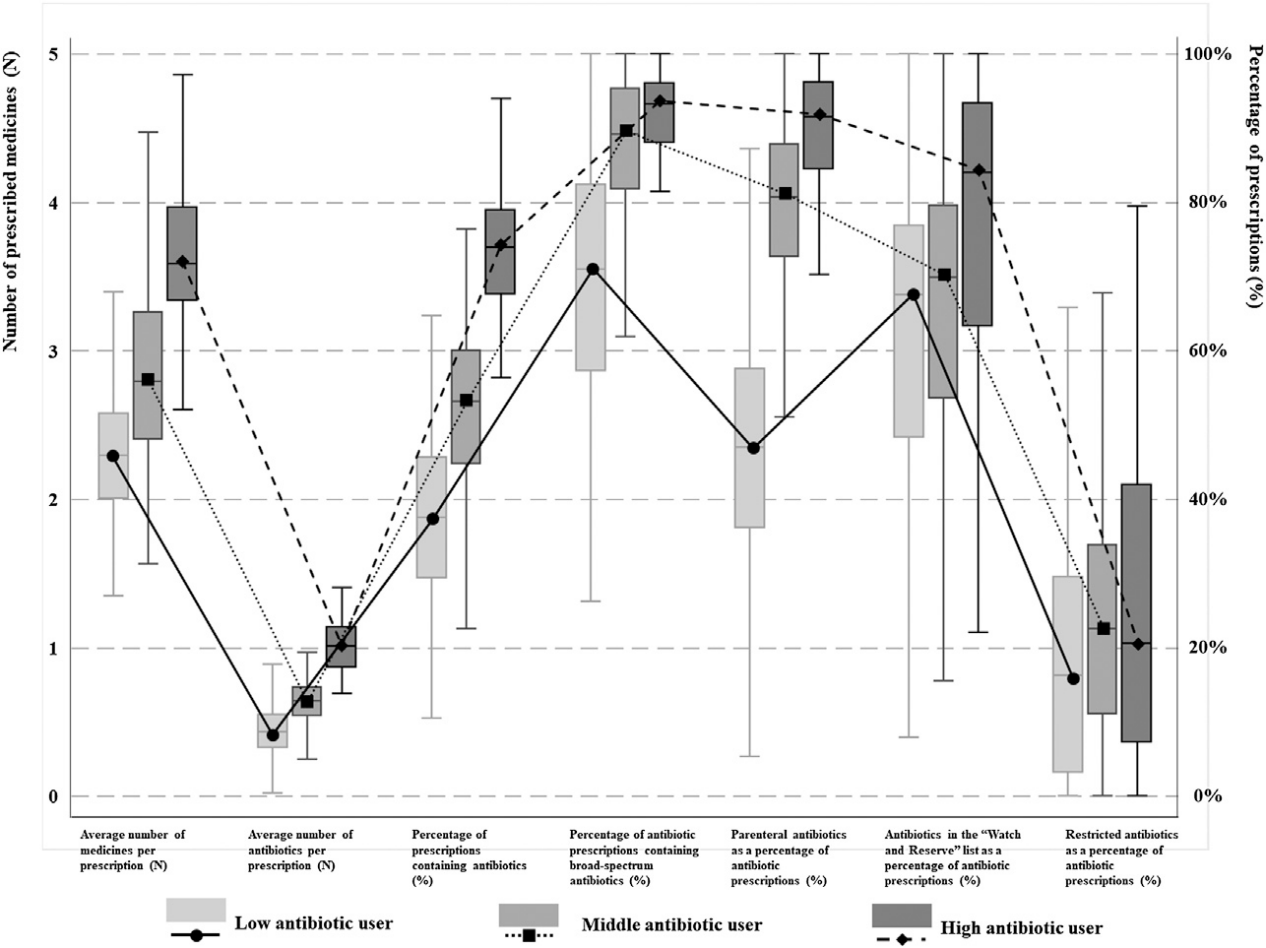
Antibiotic prescribing patterns among physicians in different groups. Three different kinds of physician’s antibiotic prescribing patterns were identified. Low, medium and high antibiotic users were classified based on seven indicators regarding to rational use of antibiotics and was presented as different lines. The means and quartiles of different patterns of different indicators were showed as boxes.

The latent profile analyses (Supplementary Table S1) identified three distinctive groups of antibiotic prescribers: 28.49% identified as low users, 51.18% as medium users and 20.33% as high users (Figure 1). The low antibiotic prescribing group was characterized by the lowest values on all of the seven indicators in comparison with the other two groups, despite a lack of statistical significance in one indicator (AWaRe) between the low and medium user groups. The high antibiotic prescribing group further distinguished itself from the medium user group through higher values on these indicators except for prescriptions of restricted antibiotics imposed by the provincial government (Table 1).

The 20.33% high antibiotic prescribers contributed to 23.56% of prescribed medicines, 24.48% of prescribed antibiotics, 26.27% of broad-spectrum antibiotics, 27.84% of parenteral administered antibiotics, 23.65% of government-restricted antibiotics, and 27.36% of antibiotics in the WHO “Watch and Reserve” list.

### Factors Associated With Antibiotic Prescribing Patterns

The 458 questionnaire respondents had an average age of 43.5 years (SD = 9.3) and 72% were male. Only 38.2% obtained a university degree. The vast majority worked in a rural setting (78.0%) and had an annual household income of less than 80,000 yuan (79.5%). About 48% of the respondents worked as a general practitioner. Slightly more than half (51.1%) had a junior professional title. The average knowledge score of the respondents sat in the middle range even though more than 75% reported attending continuing education on antibiotics (Table 2).

**TABLE 2:** Characteristics of questionnaire respondents with different prescribing patterns.

Characteristics	Overall	Low antibiotic user	Medium antibiotic user	High antibiotic user	*p* ^a^
Number of physicians (*N*, %)	458 (100%)	124 (27.07%)	243 (53.06%)	91 (19.87%)	—
Sociodemographic					
Age (Mean ± Standard deviation)	43.53 ± 9.31	45.02 ± 10.13	43.12 ± 9.02	42.62 ± 8.73	0.257
Gender					0.003
Male (*N*, %)	330 (100%)	76 (23.03%)	180 (54.55%)	74 (22.42%)	
Female (*N*, %)	128 (100%)	48 (37.50%)	63 (49.22%)	17 (13.28%)	
Educational qualification					0.018
High school and below (*N*, %)	42 (100%)	8 (19.05%)	26 (61.90%)	8 (19.05%)	
Diploma and associate degree (*N*, %)	241 (100%)	53 (21.99%)	135 (56.02%)	53 (21.99%)	
University degree (*N*, %)	175 (100%)	63 (36.00%)	82 (46.86%)	30 (17.14%)	
Annual household income (Chinese yuan ¥)					<0.001
<40,000 (*N*, %)	132 (100%)	25 (18.94%)	74 (56.06%)	33 (25.00%)	
40,000 ∼ (*N*, %)	232 (100%)	55 (23.71%)	130 (56.03%)	47 (20.26%)	
80,000 ∼ (*N*, %)	70 (100%)	27 (38.57%)	33 (47.14%)	10 (14.29%)	
≥120,000 (*N*, %)	24 (100%)	17 (70.83%)	6 (25.00%)	1 (4.17%)	
Professional practice					
Facility					<0.001
Urban community health center (*N*, %)	101 (100%)	55 (54.46%)	33 (32.67%)	13 (12.87%)	
Rural township health center (*N*, %)	357 (100%)	69 (19.33%)	210 (58.82%)	78 (21.85%)	
Years of practice (Mean ± Standard deviation)	16.54 ± 10.01	16.01 ± 10.30	16.52 ± 10.14	17.31 ± 9.17	0.520
Sub-specialty					0.025
General practice (*N*, %)	219 (100%)	67 (30.59%)	101 (46.12%)	51 (23.29%)	
Internal medicine (*N*, %)	117 (100%)	24 (20.51%)	73 (62.39%)	20 (17.09%)	
Surgery (*N*, %)	56 (100%)	10 (17.86%)	34 (60.71%)	12 (21.42%)	
Others (*N*, %)	66 (100%)	23 (34.85%)	35 (53.03%)	8 (12.12%)	
Professional title					0.017
Junior (*N*, %)	234 (100%)	50 (21.37%)	133 (56.84%)	51 (21.79%)	
Middle (*N*, %)	176 (100%)	53 (30.11%)	90 (51.14%)	33 (18.75%)	
Senior (*N*, %)	47 (100%)	21 (44.68%)	19 (40.43%)	7 (14.89%)	
Antibiotic training					0.622
Yes (*N*, %)	346 (100%)	91 (26.30%)	183 (52.89%)	72 (20.81%)	
No (*N*, %)	112 (100%)	33 (29.46%)	60 (53.57%)	19 (16.96%)	
Antibiotic knowledge (mean ± SD, range: 0–11)	6.16 ± 1.49	6.54 ± 1.35	6.01 ± 1.52	6.01 ± 1.47	0.002
Antibiotic attitudes (mean ± SD)					
Complacency (range: 0–8)	6.42 ± 1.40	6.32 ± 1.36	6.53 ± 1.36	6.23 ± 1.57	0.172
Fearful of adverse events (range: 0–12)	7.72 ± 2.00	7.85 ± 1.85	7.81 ± 2.01	7.30 ± 2.14	0.078
Ignorance of antibiotic resistance (range: 0–16)	11.59 ± 1.69	11.46 ± 1.78	11.56 ± 1.69	11.86 ± 1.52	0.210
Responsibility avoidance (range: 0–28)	9.08 ± 2.72	8.97 ± 2.64	9.14 ± 2.78	9.07 ± 2.67	0.708
Indifference to changes (range: 0–4)	2.98 ± 0.79	3.05 ± 0.66	2.96 ± 0.83	2.91 ± 0.86	0.741

a *p* values of Chi-square tests for categorical variables, Kruskal-Wallis equality-of-populations rank tests for continuous variables without a normal distribution, and ANOVA for continuous variables with a normal distribution.

Of the questionnaire respondents, 27.07% were classified as low antibiotic users, compared with 53.06% medium users and 19.87% high users. Those who were male (*p* = 0.003), had a lower educational qualification (*p* = 0.018), lived with a lower household income (*p* < 0.001), worked in rural facilities (*p* < 0.001), had a junior professional title (*p* = 0.017), and had a lower knowledge score (*p* = 0.002) were more likely to be in the high user group. There were also significant differences in the antibiotic prescribing patterns across sub-specialties (*p* = 0.025) (Table 2).

The multinomial logistic regression analyses confirmed that knowledge, attitudes, clinical experiences, household income and workplace settings were significant predictors of the antibiotic prescribing patterns after adjustments for variations in other variables (Table 3).

**TABLE 3:** Multinomial logistic regression of physician'|’s antibiotic prescribing patterns.

Variable	Medium antibiotic user		High antibiotic users	
	Relative risk ratio^a^ (95% confidence interval)	*p*	Relative risk ratio^a^ (95% confidence interval)	*p*
Sociodemographic				
Age	0.959 (0.920, 1.001)	0.055	**0.942 (0.891, 0.995)**	**0.032**
Female gender	1.217 (0.621, 2.386)	0.566	0.864 (0.37, 2.015)	0.734
Educational qualification	0.733 (0.453, 1.185)	0.204	0.749 (0.418, 1.342)	0.331
Annual household income	**0.688 (0.498, 0.952)**	**0.024**	**0.521 (0.339, 0.800)**	**0.003**
Professional practice				
Rural facility	**4.275 (2.205, 8.285)**	**<0.001**	**4.296 (1.826, 10.105)**	**0.001**
Years of practices	1.026 (0.995, 1.059)	0.104	**1.053 (1.010, 1.097)**	**0.015**
Sub-speciality				
General practice	Ref	—	Ref	—
Interal medicine	1.339 (0.704, 2.546)	0.374	0.699 (0.319, 1.533)	0.372
Surgery	1.263 (0.528, 3.020)	0.599	0.762 (0.273, 2.123)	0.603
Others	0.600 (0.264, 1.367)	0.224	0.334 (0.111, 1.010)	0.052
Professional title				
Junior	Ref	—	Ref	—
Middle	1.128 (0.621, 2.048)	0.694	1.149 (0.547, 2.414)	0.714
Senior	1.356 (0.501, 3.668)	0.549	1.735 (0.486, 6.192)	0.396
Antibiotic training	0.903 (0.506, 1.611)	0.729	1.050 (0.508, 2.172)	0.894
Knowledge score above mean	**0.440 (0.246, 0.785)**	**0.005**	**0.468 (0.234, 0.935)**	**0.031**
Attitudes scores above mean				
Complacency with satisfied patients	**2.618 (1.107, 6.191)**	**0.028**	1.935 (0.710, 5.272)	0.197
Fearful of adverse events	1.102 (0.618, 1.965)	0.743	0.822 (0.414, 1.632)	0.575
Ignorance of antibiotic resistance	0.831 (0.212, 3.250)	0.790	3.068 (0.292, 32.195)	0.350
Responsibility avoidance	1.223 (0.214, 6.976)	0.821	1.051 (0.125, 8.817)	0.964
Indifference to changes	**0.416 (0.198, 0.873)**	**0.020**	**0.401 (0.171, 0.943)**	**0.036**

a Low antibiotic user group as reference; Bold indicates statistical significance (*p* < 0.05).

The respondents with a higher than average knowledge score were less likely to be assigned into the medium (Relative Risk Ratio (RRR) = 0.440, *p* = 0.005) or high (RRR = 0.468, *p* = 0.031) antibiotic user groups as compared with the odds of low antibiotic user group. Similarly, those who reported lower indifference to changes were also less likely to be assigned into the medium (RRR = 0.416, *p* = 0.020) or high (RRR = 0.401, *p* = 0.036) antibiotic user groups. However, the respondents with lower complacency to satisfy patients were more likely to be assigned into the medium antibiotic user group only (RRR = 2.618, *p* = 0.028) as compared with the odds of low antibiotic user group.

The respondents who worked in a rural facility were more likely than their urban counterparts to be assigned into the medium (RRR = 4.275, *p* < 0.001) or high (RRR = 4.296, *p* = 0.001) antibiotic user groups as compared with the odds of low antibiotic user group. The odds of being assigned into the medium (RRR = 0.688, *p* = 0.024) and high (RRR = 0.521, *p* = 0.003) antibiotic user groups decreased with household income. The respondents with an older age had a slightly lower odds of being assigned into the high antibiotic user group (RRR = 0.942, *p* = 0.032). But longer years of practice slightly increased the odds of being assigned into the high antibiotic user group (RRR = 1.053, *p* = 0.015).

## Discussions

### Main Findings

Excessive use of antibiotics in primary care in Hubei of China is evident. The mean percentage of prescriptions involving antibiotics issued by the surveyed physicians in this study reached 52.19% (SD = 17.20%), much higher than the maximal level of 30% as recommended by the WHO (World Health Organization, 2006). Of the antibiotic prescriptions, an average of 71.92% (SD = 21.42%) were administered through parenteral injections. This forms a sharp contrast with the low level use (0.001%–6.75%) of parenteral route for antibiotics in outpatient settings in Europe (Coenen et al., 2009). The high percentage (67.74%) of antibiotic prescriptions involving antibiotics listed in the WHO “Watch and Reserve” list is also concerning. The WHO AWaRe system recommends at least 60% of prescribed antibiotics in the “Access” list, instead of the “Watch and Reserve” list, to cope with the problem of antibiotic resistance (World Health Organization, 2015).

About 20.33% of the prescribers in primary care were identified as high users of antibiotics in this study, compared with 51.18% medium users and 28.49% low users. This is a result of the combined effect of the seven prescribing indicators. The high user group contributed disproportionally across all the seven indicators. Previous studies usually classify high antibiotic prescribers using a single indicator (Jones et al., 2015; Aabenhus et al., 2017; Pouwels et al., 2018; Schmidt et al., 2018; Jung et al., 2019; Schwartz et al., 2019).

This study shows that great variation in primary care physicians’ antibiotic prescribing patterns in Hubei of China, which is shaped not only by the knowledge and attitudes of the prescribers, but also by their personal circumstances. High levels of antibiotic knowledge and attitudes in favor of practice changes are associated with low use of antibiotics. But lower household income and rural facilities are associated with high use of antibiotics.

### Comparison to Other Studies

#### Primary Care Antibiotic Prescribing

It seems that antibiotic prescribing in primary care in Hubei declined over time. The percentage of prescriptions containing antibiotics dropped from 68% in 2011 (Yang et al., 2012) to the level of 52% in 2018 as revealed in the study.

Despite the decline, irrational antibiotic prescribing remains a serious issue of concern. The use of parenteral route for antibiotics is still very high at an average level of 71.92% as a proportion of antibiotic prescriptions, despite a slight decline in comparison with the level (84%) five years ago (Yang et al., 2014). The high use of parenteral route is believed to be associated with the financial strategy to compensate for the loss of profit margins on sales of medicines (Li et al., 2017).

The domination of broad-spectrum antibiotics in antibiotic prescribing in Hubei of China is comparable to findings of other studies. A study monitoring antibiotic sales in primary cares in Hubei showed that broad-spectrum antibiotics were increasingly used in recent years, rising from 74.87% as a proportion of antibiotic sales in 2012 to 85.69% in 2017 (Zhang et al., 2019). This is not unique to China. A survey on 28 European countries showed that broad-spectrum antibiotic contributed to over 80% of antibiotic use in 22 countries (Borg and Camilleri 2019).

However, ignorance of the WHO AWaRe list in Hubei of China deserves increasing policy attention. There is serious under-use of the antibiotics in the WHO “Access” list in Hubei. The mean percentage of antibiotic prescriptions covered in the WHO “Access” list did not exceed 33% in the primary care participants in this study. This level is very low compared with the percentage of 60.17%–63.29% of “Access” antibiotics prescribed in primary care in the United Kingdom over the period from 2011 to 2017 (Budd et al., 2019). Although the mean percentage of government restricted antibiotics in antibiotic prescriptions is low at 23.52% in this study, it may be an outcome of the more relaxed policy of the provincial government (Hubei Provincial Health Committee, 2018). Similar to this study, previous studies also revealed a high level of compliance with the regional government list of restricted antibiotics (Tang et al., 2018). The effectiveness of the regional list of restricted antibiotics imposed by the government warrants further assessment.

#### Antibiotic Prescribing Patterns of Primary Care Physicians

In this study, three distinctive groups of prescribers were identified through the LPA. Great variations in the seven indicators across the three groups were revealed. The gap in the percentage of prescriptions containing antibiotics reached 2.02 times between the high and low user groups (74.43% vs. 36.76%). Despite a shortage of studies comparing individual prescribers, many existing studies point to the great variations in antibiotic prescribing across facilities and regions (Curtis et al., 2018; Edelstein et al., 2017; Goossens et al., 2005; Jung et al., 2019; Klein et al., 2018; Mölter et al., 2018; Schwartz et al., 2019; Versporten et al., 2014). The European Surveillance of Antimicrobial Consumption (ESAC) project found that physicians in France used 3.20 times of antibiotics compared with those in the The Netherlands (Goossens et al., 2005). In the United Kingdom, clinical guidelines were developed to reduce the use of trimethoprim for urinary tract infections. However, a nearly two-times gap was found in primary care in the use of trimethoprim as a proportion of nitrofurantoin and trimethoprim combined for urinary tract infections (Croker et al., 2018).

#### Factors Associated With Antibiotic Prescribing Patterns

This study found that knowledge and attitudes are significant predictors of antibiotic prescribing patterns in primary care, which is consistent with findings of previous studies (Lopez-Vazquez et al., 2012; Rodrigues et al., 2013). Good knowledge is the foundation of potential behavioral changes. But motivation is critical for translating knowledge into practice. Evidence from this study and others (Liu et al., 2019c) show that higher motivation to change is associated with less antibiotic use in primary care. However, this study found that complacency to satisfy patients does not seem to fuel antibiotic prescribing as concluded in a systematic review (Lopez-Vazquez et al., 2012). We found that primary care physicians with lower complacency to satisfy patients are more likely to be medium antibiotic users, but not high users. This may be associated with the national culture of China: reluctance to go extremes (Currie et al., 2014; Goossens et al., 2005). In addition, mistrust between physicians and patients is prevalent in China (Chan 2018).

Prescribing behaviors can also be shaped by work and policy environments (Goossens et al., 2005), as well as personal circumstances (Jung et al., 2019; Schwartz et al., 2019). This study confirms the findings of previous studies (Liu et al., 2019a), showing that physicians in rural primary care facilities are more likely to be high antibiotic users than their urban counterparts. Rural patients in China are usually exposed to poorer sanitary environment and have limited education and higher expectations on antibiotics (Reynolds and McKee, 2011; Zhang et al., 2016).

Household income was found to be a significant predictor of high use of antibiotics in primary care. This is not surprising given that prescribing can bring financial gains to the prescribers in the Chinese health system. Although primary care workers are no longer able to make a profit margin on sales of medicines, a service fee and charge for disposable syringes can still be collected (Li et al., 2017). Perverse financial incentives have been widely believed to be the main driver of antibiotic abuse in China (Currie et al., 2014; Xue et al., 2019). Prescribers with low household income are particularly vulnerable to the perverse incentives.

### Implications

To address AMR, many countries and institutions have established a surveillance system monitoring the use of antibiotics. The LPA adopted in this study can help identify high antibiotic prescribers. Such a strategy should only be used for targeted interventions for continuing quality improvement. It is inappropriate to punish those deemed “high users” because the major drivers of high use of antibiotics come from the system, not the individuals (Liu et al., 2019a).

To curb overuse of antibiotics in primary care in China, multiple strategies need to be taken. Both restrictive and persuasive measures should emphasize on the overall reduction of antibiotic prescriptions, as well as the limited use of broad-spectrum, parenteral administrated, and the WHO “Watch and Reserve” antibiotics. In addition, the regional list of restricted antibiotics imposed by the government should be better aligned with the WHO AWaRe list. Significant increase in governmental budget support to primary care is needed to break the perverse financial incentives (Yang et al., 2015).

In addition, several potential initiatives could be considered to help change physicians irrational antibiotic prescribing patterns, which have been highlighted in existing evidence (Dyar et al., 2016; Godman et al., 2020), including education and training, improvement of physician communication skills, introduction of guidelines and clinical decision support systems and implementation of delayed prescribing policy.

### Strength and Limitations

Extensive studies have been undertaken to explore variations in antibiotic prescribing practices (Curtis et al., 2018; Edelstein et al., 2017; Goossens et al., 2005; Klein et al., 2018; Mölter et al., 2018; Versporten et al., 2014). But very few, if any, have attempted to identify high-profile users of antibiotics. The LPA technique provides an instrument to classify antibiotic prescribers using multiple indicators. This is important because different prescribing indicators examine the issue through different angles. For example, a high antibiotic prescriber does not necessarily always use more “restricted” antibiotics or parenteral route, and vice versa.

This study identified factors associated with antibiotic prescribing patterns based on an extended knowledge-attitude-practice theory, a framework commonly used for exploring behaviors of health practitioners (Rodrigues et al., 2013). Validated scales were adopted to measure antibiotic knowledge and the five sub-dimensions of attitudes toward antibiotic prescribing. A lack of well-validated instruments for measuring knowledge and attitudes in previous studies was commonly criticized (Alumran et al., 2012). Contextual factors were also considered in this study. Empirical evidence shows that personal circumstances can shape the behavioral patterns of medical practitioners (Lopez-Vazquez et al., 2012; Rodrigues et al., 2013).

There are several limitations to be mentioned. The study was conducted in Hubei province using a cross-sectional design. This does not allow us to draw causal conclusions. The results should not be generalized to other regions. Instead, replications of the study in other regions using the proposed approach are advised. Further studies also need to consider risk-adjustments, in particular in hospital settings where patient conditions vary considerably. This study was not able to adjust the results for variations in patient conditions, simply because such data and risk-adjustment tools were not available.

## Conclusion

In primary cares, over-use of antibiotics is prevalent in Hubei of China, particularly in the prescribing of broad-spectrum, parenteral administrated and restricted antibiotics. Great individual variation in antibiotic prescribing patterns exists. Those who are deemed high users contribute disproportionally to the inappropriate use of antibiotics. Prescribers worked in a rural setting and those with insufficient knowledge, low motivations for behavioral changes, and low household income are more likely to be high users. To curb physician irrational use of antibiotics in primary care, multiple strategies should to be taken, including developing a surveillance system comprehensively monitoring and analyzing physician antibiotic practices, training and education emphasizing on broad-spectrum and parenteral administrated antibiotic use and sufficient financial support for primary cares to break incentives.

## Data Availability Statement

The raw data supporting the conclusions of this article will be made available by the authors, without undue reservation.

## Ethics Statement

The studies involving human participants were reviewed and approved by This study has been approved by the Ethics Committee of Tongji Medical College, Huazhong University of Science and Technology (No: IORG 0003571). The patients/participants provided their written informed consent to participate in this study.

## Author Contributions

CXL designed the project and participated in the collection and interpretation of data. DW contributed to the acquisition, analysis and interpretation of data and drafted the manuscript. CJL participated in data analysis, interpretation of results, and writing of the manuscript. XZ participated in the cleaning and interpretation of data. All authors have read and approved the final version of the article.

## Funding

This study was funded by the National Natural Science Foundation of China (grant no. 71373092 & 71904053). The funding body played no part in the study design, collection, analysis and interpretation of data, writing of the manuscript or the decision to submit the manuscript for publication.

## Conflict of Interest

The authors declare that the research was conducted in the absence of any commercial or financial relationships that could be construed as a potential conflict of interest.

## Contribution to the Field

Inappropriate and over-prescriptions of antibiotics is commonly witnessed worldwide, contributing to antibiotic resistance and threatening global health and economic development. To address this issue, promoting physician’s rational prescribing of antibiotics is significant. However, our understanding about antibiotic prescribing patterns is quite limited, despite great variations existed in physician’s antibiotic prescribing practices which indicated huge potential for improvement. Therefore, this study adopted seven indicators to comprehensively assess physician’s antibiotic use behaviors and applied a two-level latent profile analysis to objectively classify physicians into different antibiotic use patterns. In addition, the identified physician’s prescribing patterns were further linked with their knowledge, five sub-kinds of attitudes and personal characteristics to diagnose why physicians showed different antibiotic use patterns. Based on the current study, physicians were classified as three different antibiotic use patterns and those who are deemed as high users contribute disproportionally to the inappropriate use of antibiotics. Higher antibiotic use pattern was linked with insufficient knowledge, indifference to changes, complacency with satisfied patients, limited household income and rural setting of the prescribers. The classification technique used in the current study can help identify high antibiotic prescribers and explore the underlying determinants of their behavioral patterns, informing more targeted interventions generated for improvement.

## Abbreviations

**Abbreviations**: AMR, antimicrobial resistance; WHO, World Health Organization; AWaRe, Access, Watch and Reserve; ATC, anatomical therapeutic chemical; LPA, latent profile analysis; MLR, Maximum likelihood parameter estimates with standard errors; BIC, Bayesian Information Criterion; SABIC, Sample-size Adjusted BIC; VLMR-LRT, Vuong-Lo-Mendell-Rubin Adjusted Likelihood Ratio Test; cmP, Correct Model Probability; ANOVA, one-way analysis of variance; RRR, Relative Risk Ratio; ESAC, European Surveillance of Antimicrobial Consumption; SD, standard deviation.
