# Supplementary material for: Identifying Antibiotic Prescribing Patterns Through Multi-Level Latent Profile Analyses: A Cross-Sectional Survey of Primary Care Physicians
Source: Front Pharmacol. 2020 Nov 11;11:591709. doi: 10.3389/fphar.2020.591709 (PMC7748108; doi:10.3389/fphar.2020.591709)
Supplement: Supplementary file 1 [file Table1_v1.docx]

| **Appendix Table S1: Fit statistics of latent profile models with different numbers of classified groups*** | | | | | | |
| --- | --- | --- | --- | --- | --- | --- |
| **Potential prescribing patterns** | **BIC** | **SABIC** | **VLMR-LRT** | **cmP** | **Entropy** | **Proportion of prescribers in the smallest group** |
| 1 group | 32.119 | -12.323 | - | 0.000 | - | - |
| 2 groups | -927.458 | -1000.470 | p=0.5809 | 0.000 | 0.865 | 42.47% |
| 3 groups | -1265.276 | -1366.857 | p=0.4652 | 0.000 | 0.865 | 20.98% |
| 4 groups | -1465.142 | -1595.294 | p=0.1614 | 1.000 | 0.890 | 2.85% |
| 5 groups** | - | - | - | - | - | - |
| *BIC: Bayesian Information Criterion; SABIC: Sample-size adjusted BIC; VLMR-LRT: Vuong-Lo-Mendell-Rubin adjusted likelihood ratio test; BF: Bayes Factor; cmP: correct model probability;  **The five-group pattern was dropped due to failure in the model identification test | | | | | | |
